# Rapid Induction and Maintenance of Virus-Specific CD8^+^ T_EMRA_ and CD4^+^ T_EM_ Cells Following Protective Vaccination Against Dengue Virus Challenge in Humans

**DOI:** 10.3389/fimmu.2020.00479

**Published:** 2020-03-24

**Authors:** Nancy Graham, Phil Eisenhauer, Sean A. Diehl, Kristen K. Pierce, Stephen S. Whitehead, Anna P. Durbin, Beth D. Kirkpatrick, Alessandro Sette, Daniela Weiskopf, Jonathan E. Boyson, Jason W. Botten

**Affiliations:** ^1^Department of Microbiology and Molecular Genetics, Larner College of Medicine, University of Vermont, Burlington, VT, United States; ^2^Vaccine Testing Center, Larner College of Medicine, University of Vermont, Burlington, VT, United States; ^3^Department of Medicine, Larner College of Medicine, University of Vermont, Burlington, VT, United States; ^4^Laboratory of Infectious Diseases, National Institute of Allergy and Infectious Diseases, National Institutes of Health, Bethesda, MD, United States; ^5^Bloomberg School of Public Health, Johns Hopkins University, Baltimore, MD, United States; ^6^Division of Vaccine Discovery, La Jolla Institute for Immunology, La Jolla, CA, United States; ^7^Department of Medicine, University of California, San Diego, San Diego, CA, United States; ^8^Department of Surgery, Larner College of Medicine, University of Vermont, Burlington, VT, United States

**Keywords:** dengue, vaccine, CD8, CD4, T_EMRA_, protective immunity, memory, human challenge

## Abstract

Dengue virus (DENV) is a mosquito-borne flavivirus that causes serious human disease. The current lack of an effective vaccine to simultaneously protect against the four serotypes of DENV in seronegative individuals is a major unmet medical need. Further, the immunological basis for protective immunity in the setting of DENV infection or vaccination is not fully understood. Our team has developed a live attenuated tetravalent dengue virus vaccine that provides complete protection in a human model of dengue virus challenge. The goal of this study was to define, in the context of protective human vaccination, the quality of vaccine-induced DENV-specific CD8^+^ and CD4^+^ T cells and the temporal dynamics associated with their formation and maintenance. Multifunctional, DENV-specific CD8^+^ and CD4^+^ T cells developed 8–14 days after vaccination and were maintained for at least 6 months. Virus-specific CD8 T^+^ cells were a mixture of effector memory T cells (T_EM_) and effector memory T cells re-expressing CD45RA (T_EMRA_), with T_EM_ cells predominating until day 21 post-vaccination and T_EMRA_ cells thereafter. The majority of virus-specific CD4^+^ T cells were T_EM_ with a small fraction being T_EMRA_. The frequency of virus-specific CD8^+^ and CD4^+^ T cells were further skewed to the T_EMRA_ phenotype following either a second dose of the tetravalent vaccine or challenge with a single serotype of DENV. Collectively, our study has defined the phenotypic profile of antiviral CD8^+^ and CD4^+^ T cells associated with protective immunity to DENV infection and the kinetics of their formation and maintenance.

## Introduction

Dengue virus (DENV), a mosquito-borne flavivirus, is the most prevalent cause of arboviral disease in humans. Nearly half of the world's population is at risk for DENV disease and each year there are ~390 million cases in over 120 countries (1). There are four distinct serotypes of DENV (DENV1-4) and each is capable of causing the full range of clinical disease, from asymptomatic infection to death from DENV disease (2). While many individuals experience a relatively undifferentiated febrile illness, others develop severe clinical syndromes (dengue hemorrhagic fever and dengue shock syndrome) that are associated with severe thrombocytopenia and clotting disorders, as well as plasma leakage. These more severe disease syndromes are associated with increased risk of death, particularly in areas lacking sufficient medical care or in the very young or old (3). Although a vaccine for the prevention of DENV disease was recently approved by the United States Food and Drug Administration (FDA), its use is restricted to individuals 9–16 years of age with laboratory-confirmed previous dengue infection (4). Therefore, there remains a critical need for a broadly effective vaccine that protects dengue-naïve individuals.

A unique feature of DENV that complicates vaccine development is the observation that individuals, when infected with a second and different serotype of DENV, have a higher risk of severe disease and poor outcomes (5). Serotype-specific neutralizing antibodies raised following a first infection successfully protect against symptomatic infection with that serotype for life. However, non-neutralizing antibodies capable of binding other DENV serotypes can also be induced by a primary infection. These antibodies, when bound to a virus particle from a heterologous DENV serotype, are thought to predispose the heterologous virus for “enhanced” entry and replication in target cells when the individual is infected subsequently with this heterologous serotype (6). Thus, antibody-dependent enhancement (ADE) of infection is thought to be a key mechanism by which heterotypic, non-neutralizing antibodies may increase the risk of severe clinical disease and must be accounted for in vaccination strategies (7).

Because of the risk of developing ADE, the major global concern surrounding dengue vaccine development is that vaccination may create gaps in simultaneous coverage to all four serotypes. These gaps may emerge either when the initial vaccine series does not sufficiently prompt initial protective immunity against all four serotypes and/or due to waning coverage over time to one or more serotypes (8). In either scenario, partial protection from vaccination may expose a vaccinated individual to a risk of severe and/or life-threatening dengue disease if infected with the serotype for which there is a gap in coverage. It is possible that this risk may be higher due to vaccination than for those who were never vaccinated when the vaccine induces only partial protection. Indeed, this very concern has emerged following introduction of the Dengvaxia® vaccine into endemic areas (9). This tetravalent vaccine is constructed on the non-structural backbone of the 17D Yellow Fever vaccine. It contains the structural membrane (M) and envelope (E) proteins of DENV and the structural capsid (C) and non-structural proteins of Yellow Fever virus. Early studies of Dengvaxia® in humans and non-human primates suggested incomplete immunity (10) and imbalanced antibody responses across serotypes in early human trials (11–14). Field data now confirms that individuals who are dengue-naïve when they received Dengvaxia® have a higher risk of hospitalization with subsequent dengue infection compared with unvaccinated individuals (9, 15, 16). A possible contributing mechanism to poor protection may be the vaccine's lack of non-structural DENV proteins, which have been demonstrated to be the predominant target of dengue-specific CD4^+^ and CD8^+^ T cell responses (17–21).

Members of our team developed the NIH dengue live attenuated tetravalent vaccine (DLAV). Constructed via reverse genetics, this vaccine encodes wild-type dengue structural and non-structural proteins and one or more 30-nucleotide deletions in the 3′ untranslated region as its core attenuation strategy (22–25). Comprehensive development over 20 years (26–31) has led to two tetravalent formulations (TV003 and TV005) that are well-tolerated with no fever, and no liver function or clotting function abnormalities. This vaccine induces neutralizing antibodies to DENV1-4 with high frequency (31, 32) and also elicits multifunctional CD8^+^ and CD4^+^ T cells to each DENV serotype (19, 20). In an effort to evaluate the protective efficacy of DLAV, we developed a controlled human model of immunization and challenge in which individuals were immunized with DLAV and challenged 6 months later with under-attenuated strains of DENV. Notably, in the setting of this controlled human infection model, DLAV immunization resulted in complete protection against DENV2 or DENV3 infection (e.g., the vaccinees did not develop viremia, rash, or neutropenia) (32) (data not shown).

At present there is an incomplete understanding of what constitutes protective immunity in the setting of DENV infection. Further, it is unknown how quickly protective immunity is established following infection or vaccination. Neutralizing antibodies certainly contribute to protection, possibly by providing sterilizing immunity to a subsequent DENV exposure. There is also evidence to suggest that antiviral CD8^+^ and CD4^+^ T cells contribute to protective immunity and abrogation of severe disease. First, in the setting of murine infection, both cell types play a direct role in protection (33–40). Second, HLA alleles associated more severe disease correlate with weak CD4^+^ and CD8^+^ T cell responses while HLA alleles associated with less severe disease correlate with more robust and multifunctional T cell responses (18, 21, 41, 42). This data collectively suggests that anti-DENV T cells contribute to protective immunity.

In the current study, our goal was to evaluate CD8^+^ and CD4^+^ T cell phenotype and function following protective human vaccination with DLAV. In particular, we studied two cohorts: one that was vaccinated with DLAV and then boosted 180 days later (31) and the other that was vaccinated with DLAV and then challenged 180 days later with DENV2Δ30 (Tonga/74), an American genotype DENV2 strain that was isolated during an outbreak of DENV in the Kingdom of Tonga in 1974 and is heterotypic to the parent of the vaccine strain (DENV2 strain New Guinea C) (32, 43). Notably, all individuals in the latter study were completely protected from DENV challenge; DENV2 challenge virus was not detected in any vaccinated subject either by infectious virus isolation or by RT-PCR (32). Here, we describe the natural history of DLAV-induced CD8^+^ and CD4^+^ T cell formation and maintenance and the phenotypic attributes of these T cell subsets.

## Materials and Methods

### Study Participants

Subjects in this study were participants of phase I studies to evaluate the safety and immunogenicity of the tetravalent live attenuated dengue vaccine TV003 trial CIR268 (Clinicaltrials.gov NCT01072786) (31) and trial CIR287 (Clinicaltrials.gov NCT02021968) (32). Based on the availability of high quality cryopreserved peripheral blood mononuclear cells (PBMC), we were able to evaluate T cell responses from 16 CIR 268 donors (*n* = 6 who were immunized with a single dose of TV003; *n* = 10 who were immunized with TV003 and then given a second dose 180 days later) and 8 CIR287 donors. All subjects were serologically confirmed as flavivirus-naïve at the time of immunization. Studies were approved by the Institutional Review Boards at the University of Vermont and Johns Hopkins University. Informed consent was obtained in accordance with federal and international regulations (21CFR50 and ICHE6). External monitoring was performed by National Institute of Allergy and Infectious Diseases Data Safety Monitoring board every 6 months.

### Clinical Sample Procurement

At study visits, blood was collected by venipuncture into serum separator tubes for analyses of viremia and serology, and into EDTA tubes for isolation of peripheral blood mononuclear cells (PBMC). Serum was frozen at −20°C until use. PBMC were isolated by Ficoll-paque density gradient separation, counted, and frozen in cell culture medium with 10% dimethyl sulfoxide (DMSO) and 40% fetal bovine serum (FBS), and cryopreserved in liquid nitrogen vapor phase.

### Vaccine (TV003) and Challenge Virus (rDEN2Δ30)

**Figure 1:**
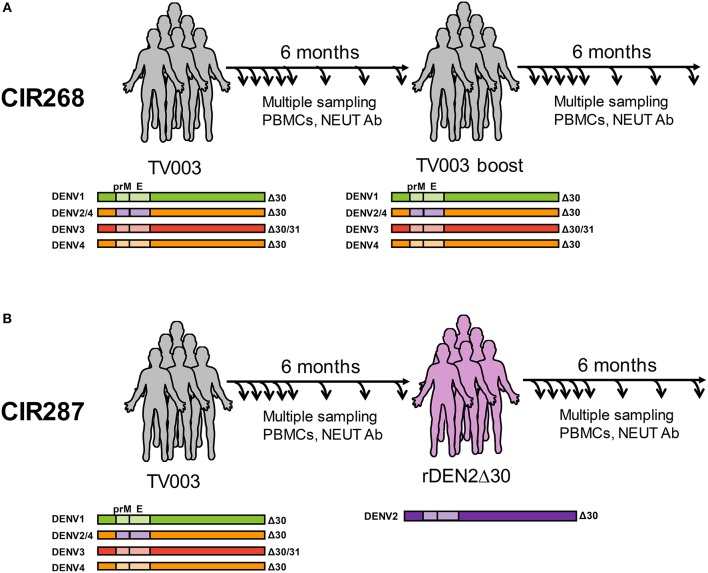
Overview of human cohorts for measurement of anti-DENV T cells following vaccination and/or challenge. **(A)** Immunization schedule of the CIR268 study. Donors received the TV003 formulation of DLAV on day 0 and were given a second dose of TV003 on day 180 post-primary vaccination. **(B)** Immunization and challenge schedule of the CIR287 study. Donors were immunized with TV003 on day 0 and were challenged with rDENV2Δ30 (Tonga/74) on day 180 post-vaccination. For both studies, blood and PBMC were collected at multiple times post-vaccination or post-challenge for analysis by ELISPOT, ICS, or FRNT.

The TV003 formulation of DLAV is an admixture composed of three DENVs attenuated by deletion(s) in the 3′ untranslated region (3′UTR): rDENV1Δ30, rDENV3Δ30/31, and rDENV4Δ30, and a fourth component that is a chimeric virus with the prM and E proteins of DENV2 NGC (New Guinea C strain) exchanged for DENV4 in the rDENV4Δ30 genome (rDENV2/4Δ30) (illustrated in Figure 1) (31, 32). Each donor received 10^3^ PFU of each DENV strain via subcutaneous inoculation. The challenge strain rDEN2Δ30 is a recombinant virus derived from the DENV2 Tonga/74 wild-type virus (43), a different genotype than DEN2 NGC. Study participants received 10^3^ PFU of this challenge virus via subcutaneous injection.

### DENV Epitopes

To facilitate detection of DENV-specific T cell responses irrespective of HLA types and DENV serotypes in various immunological contexts where only small amounts of blood are available, we combined previously identified DENV epitopes into a single peptide pool [megapool (MP)] that was used for T cell stimulation. DENV MPs were generated for both CD4^+^ and CD8^+^ T cells, and consisted of 180 and 268 peptides, respectively (see Table S1 for a list of these peptides). Peptides were pooled, lyophilized, and resuspended in DMSO to form a master mix, which was then used to stimulate T cells *ex vivo*. DENV CD4 and CD8 MPs account for 62 and 90% of the IFN-γ response in Sri Lankan and Nicaraguan cohorts, respectively, and have been validated in different geographical locations supporting their global applicability (18, 21, 42, 44).

### *Ex vivo* IFN-γ Enzyme-Linked Immunosorbent Spot (ELISPOT) Assay

Flat-bottom, 96-well nitrocellulose plates (Immobilon-P; Millipore) were pre-coated overnight with 50 μL of anti-human IFN-γ mAb 1-D1K (1 mg/mL) (3420-3-250; Mabtech). The next day, after washing the plates three times with PBS, 2 × 10^5^ PBMC from each donor were plated in triplicate with either 0.5 μL of the DENV CD8 MP (4 μg/mL), 0.5 μL DMSO (negative control), 20 μL of phytohemagglutinin (PHA [1 mg/mL]) (positive control), or 1 μL each of PMA (100 μg/mL) and ionomycin (1 mg/mL) (positive control) for 16–20 h at 37°C. Plates were then washed six times with PBS/0.05% Tween 20 and incubated with 100 μL/well of biotinylated anti-IFN-γ mAb 7-B6-1 (1 mg/mL) (3420-6-250; Mabtech) for 2 h at 37°C. After six additional washes with PBS/0.05 Tween 20, IFN-γ spots were developed by sequential incubation with Vectastain ABC peroxidase (Vector Laboratories) and 3 amino-9-ethyl carbazole solution (Sigma-Aldrich) and counted by computer assisted image analysis (ZEISS KS ELISPOT Reader). Each patient sample was tested in three replicate wells and the experimental values were expressed as mean spots/10^6^ PBMC. For each sample tested, responses to DMSO were measured (to establish background values) and subtracted from the response to the DENV CD8 MP.

### Flow Cytometry and Intracellular Cytokine Staining (ICS) Assay

PBMCs (2 × 10^6^) were cultured in the presence of DENV CD8 or CD4 MPs (1 μg/mL), DMSO (negative control), or PMA (100 ng/mL)/ionomycin (1 μg/mL) (positive control) for 2 h at 37°C. GolgiPlug (BD Biosciences) was then added and cells were incubated for an additional 4 h at 37°C. Cells were washed, and then stained with Live Dead Fixable Blue staining reagent (Thermo Fisher) at 4°C for 30 min, after which they were resuspended in staining buffer (PBS/1% human AB serum) and Brilliant Violet Staining buffer (BD Biosciences) containing surface staining antibodies and incubated at 4°C for 30 min. For intracellular staining, cells were fixed in ice-cold PBS/4% paraformaldehyde for 10 min, washed, and incubated in staining buffer at 4°C overnight. Cells were permeabilized with PBS/1% human AB serum/0.1% sodium azide/0.1% saponin, after which they were incubated with 10% human serum in permeabilization buffer, and then stained for intracellular cytokine expression at 4°C for 30 min. Flow cytometry data were collected on a LSRII flow cytometer (BD Bioscience) and analyzed with FlowJo software (Treestar). For the CD8^+^ and CD4^+^ T cell analyses, the background signal from DMSO was subtracted from the signal elicited by the DENV CD8 MP or the DENV CD4 MP.

Antibodies used in these experiments were as follows: CD3 (UCHT1), CD19 (SJ25C1), CD14 (M0Pg) from BD Biosciences, CD4 (OKT4), CD8a (RPA-T8), and CD197 (G043H7) from Biolegend, CD45RA (HI100) from Thermo Fisher Scientific, and TNF-α (Mab11) and IFN-γ (4S.B3) from eBioscience.

### Focus Reduction Neutralization (FRNT) Assay

Serum neutralizing antibody titers against DENV1-4 were determined by focus reduction neutralization test (FRNT), using the lowest serum dilution that yielded a 50% reduction in viral foci (FRNT_50_) as previously described (32). The virus strains used were DENV1 (WestPac/74), DENV2 (New Guinea C), DENV3 (Slemen/78), and DENV4 (Dominica/81).

## Results

### Human Cohorts for Measurement of Anti-DENV T Cells Following Vaccination and/or Challenge

We have previously reported on the ability of the NIH DENV tetravalent live-attenuated vaccine (DLAV) to induce DENV-specific T cells (19, 20) and neutralizing antibodies (31, 32), as well as its ability to protect against challenge with an under-attenuated strain of DENV (32). In the current study, our goal was to define the natural history of antiviral CD8^+^ and CD4^+^ T cells in the setting of protective vaccination. We leveraged T cells obtained from two vaccination studies. The first was study CIR268, where individuals were either (i) vaccinated with DLAV and followed for 180 days or (ii) vaccinated with DLAV and given a boost of DLAV 180 days later (Figure 1A). We previously reported on the immunogenicity of vaccination in these individuals relating to the formation of anti-DENV neutralizing antibodies (31). The second study was CIR287, which followed individuals that received DLAV and 180 days later were protected from challenge with DENV2Δ30 (Tonga/74) (32) (Figure 1B). Herein, we report on the phenotypic and temporal properties of DENV-specific T cells in the context of these two studies, which collectively provide models of (i) single-dose vaccination, (ii) multi-dose vaccination, or (iii) single-dose vaccination and subsequent protection against challenge.

### Natural History of DENV-Specific CD8^+^ T Cell Formation and Maintenance Following Vaccination and a Subsequent Boost

**Figure 2:**
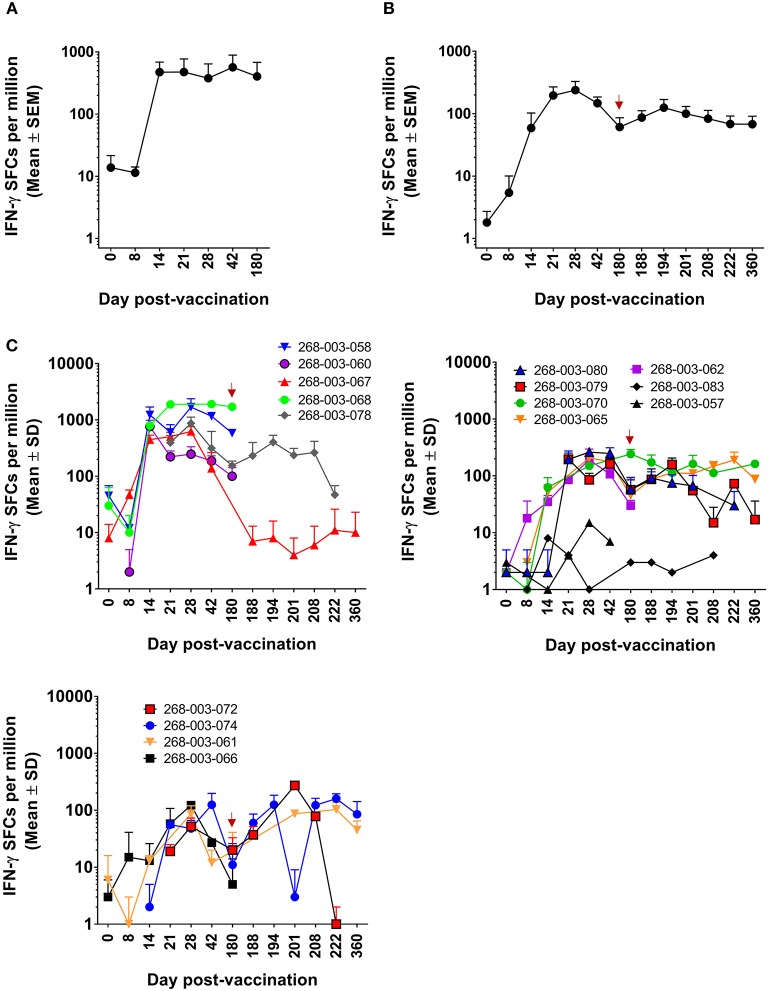
ELISPOT measurement of DENV-specific IFN-γ^+^CD8^+^ T cells following DLAV vaccination and boosting in CIR268 individuals. ELISPOT analysis was used to identify the number of IFN-γ producing CD8^+^ T cells that responded to the DENV CD8 MP following DLAV vaccination on day 0 and DLAV boosting on day 180 post-primary vaccination. Note that the time of DLAV boosting (day 180 post-primary vaccination) is shown in each graph in **(B,C)** with a red arrow. **(A)** Depicts the mean responses ± SEM for the donors that received only the primary DLAV vaccination and were followed to day 180 (*n* = 6) while **(B)** shows the mean responses ± SEM for donors that received DLAV vaccination on day 0 and a boost of DLAV on day 180 post-primary vaccination (*n* = 10). **(C)** Shows individual donor responses for all vaccinees.

We previously demonstrated that DENV-specific CD8^+^ T cells can be detected 11–13 months after DLAV vaccination (19). Using the CIR268 cohort (described in Figure 1A), we wanted to determine (i) the timing of DENV-specific CD8^+^ T cell formation and maintenance following DLAV vaccination and (ii) the impact of a second DLAV dose on the frequency and durability of these vaccine-induced CD8^+^ T cells. To ensure maximal sensitivity, we initially employed the ELISPOT assay to detect CD8^+^ T cells capable of secreting IFN-γ in response to the DENV CD8 MP, which contains the most frequently observed CD8^+^ T cell epitopes from each of the four DENV serotypes, regardless of HLA background (for further description, see Materials and Methods). As shown in Figure 2, when all donors (*n* = 6 who were immunized with a single dose of DLAV; *n* = 10 who were immunized with DLAV and then given a second dose 180 days later) were examined, DENV-specific CD8^+^ T cells first became detectable as early as 8 days post-vaccination, with most donors exhibiting their first measurable responses 14–21 days after vaccination. Peak frequencies were typically observed between 21 and 42 days post-vaccination, followed by declining responses through day 180 post-vaccination. However, responses remained detectable through this entire time period for most donors, with the exception of 268-003-067 and 268-003-068, who had undetectable responses at the day 180 post-vaccination time point. Additionally, responses were not detectable by ELISPOT at any time point for donors 268-003-057 and 268-003-083, despite the fact that both donors generated neutralizing antibodies (data not shown). This could reflect (i) a limit in the sensitivity of our ELISPOT assays to detect responses that may be present in these donors, (ii) an incompatibility between the HLA genotype of these donors and the CD8^+^ T cell epitopes included in the megapool, or (iii) that these donors failed to make CD8^+^ T cell responses to vaccination.

For the CIR268 donors who received a second dose of DLAV on day 180 after primary DLAV vaccination, we observed that DENV-specific CD8^+^ T cell frequencies either increased (*n* = 7), or decreased (*n* = 2) when compared to the levels detectable at the day 180 post-vaccination time point (Figure 2C). For those with CD8^+^ T cell expansion, maximal cell frequencies were observed between 14 and 42 days after the second dose, followed by a decline similar to what was observed following primary vaccination. The majority of vaccinees who received a second dose retained detectable antiviral CD8^+^ T cell cells through day 360 post-primary vaccination (day 180 post-boost).

### DLAV Vaccination Elicits Multifunctional CD8^+^ T Cells

**Figure 3:**
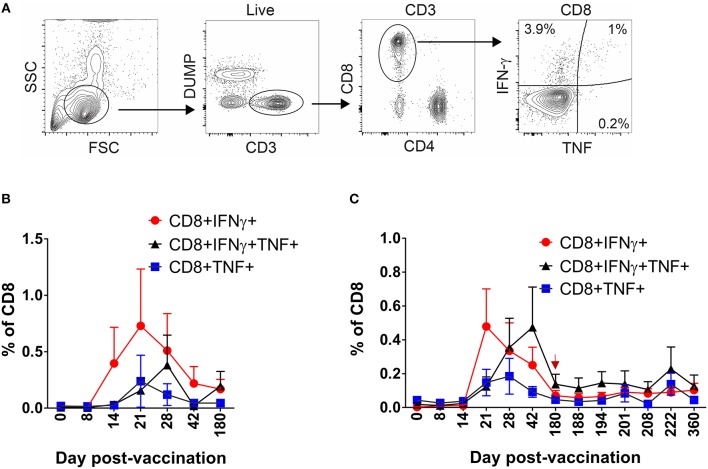
Kinetics of multifunctional CD8^+^ T cell formation and maintenance following DLAV vaccination or boost in CIR268 individuals. ICS was used to measure the frequency of CD8^+^ T cells that made IFN-γ, TNF-α, or both in response to the DENV CD8 MP following DLAV vaccination on day 0 and DLAV boosting on day 180 post-primary vaccination. The time of DLAV boosting (day 180 post-primary vaccination) is shown with a red arrow in **(C)**. **(A)** Depicts the gating strategy used for these analyses. For each patient sample, the background signal to DMSO was subtracted from the signal to the DENV CD8 MP. **(B)** Depicts the mean ± SEM responses for the donors that received only the primary DLAV vaccination and were followed to day 180 (*n* = 6) while **(C)** shows the mean responses for donors that received DLAV vaccination on day 0 and a boost of DLAV on day 180 post-primary vaccination (*n* = 10).

Having defined the kinetics and dynamics of DENV-specific CD8^+^ T cell formation and maintenance following DLAV vaccination or subsequent boosting, we next wished to characterize the phenotypic properties of these cells using the intracellular cytokine staining (ICS) assay. Specifically, PBMC from CIR268 vaccinees were stimulated with the DENV CD8 MP and the virus-specific CD8^+^ T cells within each PBMC population were assessed for their ability to produce cytokines (IFN-γ or TNF-α) as well as express memory markers (CCR7 and CD45RA). Figure 3A shows our gating scheme. As shown in Figures 3B,C, DENV-specific CD8^+^ T cells secreting IFN-γ or TNF-α became detectable 14 days after vaccination and peaked at 21 days. In contrast, the appearance of multifunctional, DENV-specific CD8^+^ T cells secreting both IFN-γ and TNF-α appeared later at day 21 post-vaccination and reached peak values 28–42 days following vaccination. Similar to the cohort-wide averaged ELISPOT results shown Figures 2A,B, the frequencies of all three populations of DENV-specific CD8^+^ T cells (those making IFN-γ, TNF-α, or both) declined thereafter until day 180 post-vaccination (Figures 3B,C). Delivering a second dose of DLAV did not lead to a significant change in the cell frequencies through the remaining 180 days following the boost (Figure 3C).

### DLAV Vaccination Elicits Dynamic DENV-Specific T_EM_ and T_EMRA_ CD8^+^ T Cell Populations

**Figure 4:**
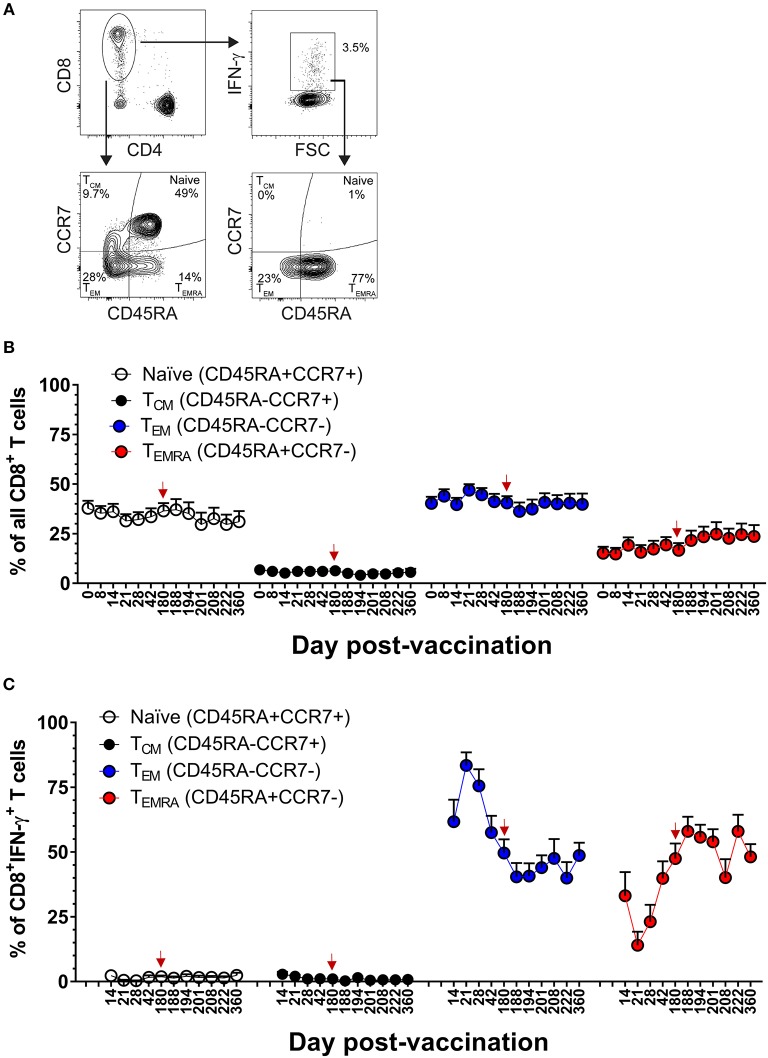
DLAV vaccination and boosting elicits dynamic DENV-specific T_EM_ and T_EMRA_ CD8^+^ T cell populations. **(A)** Depicts the gating strategy used for these analyses. **(B)** ICS was used to measure the frequency of all CD8^+^ T cells exhibiting different memory T cell phenotypes (naïve, CD45RA^+^CCR7^+^; T_CM_, CD45RA^−^CCR7^+^; T_EM_, CD45RA^−^CCR7^−^; or T_EMRA_, CD45RA^+^CCR7^−^) in CIR268 vaccinees following DLAV vaccination on day 0 and DLAV boosting on day 180 post-primary vaccination. **(C)** Shows the frequency of IFN-γ^+^CD8^+^ T cells that exhibited different memory phenotypes following stimulation with the DENV CD8 MP. Mean values ± SEM are shown (*n* = 6 that received only the primary DLAV vaccination and were only following this primary vaccination; *n* = 11 that received DLAV vaccination on day 0 and a boost of DLAV on day 180 post-primary vaccination). Note that the time of DLAV boosting (day 180 post-primary vaccination) is shown in each graph with a red arrow.

Our team has previously reported that the majority of DENV-specific memory CD8^+^ T cells induced by DLAV at 11–13 months after vaccination are of the T effector memory phenotype where some cells were re-expressing CD45RA (T_EMRA_) (CD45RA^+^, CCR7^−^) (19). The frequent sampling points in the CIR268 study provided an opportunity to map the kinetics DENV-specific memory CD8^+^ T cell formation following vaccination and boosting. Figure 4A shows our gating scheme while Figures 4B,C show memory marker expression on total CD8^+^ T cells or DENV-specific, IFN-γ^+^CD8^+^ T cells, respectively. An examination of DENV-specific CD8^+^ T cells expressing IFN-γ revealed a mixture of T effector memory (TEM) (CD45RA^−^, CCR7^−^) and T_EMRA_ on day 14 following vaccination, while no T central memory (TCM) (CD45RA^−^, CCR7^+^) cells were detected (Figure 4C). Strikingly, we noted a predominance of DENV-specific CD8^+^ T_EM_ cells at days 14–21 post-vaccination, with a peak CD8^+^ T_EM_ frequency at day 21 (~83% of IFN-γ^+^CD8^+^ T cells). Thereafter, CD8^+^ T_EM_ frequencies declined, while CD8^+^ T_EMRA_ frequencies steadily increased until day 180 post-vaccination, where the proportion of T_EM_ and T_EMRA_ IFN-γ^+^CD8^+^ T cells was similar (~50% of each). Interestingly, following the second dose of DLAV at day 180 post-vaccination, CD8^+^ T_EMRA_ cells continued to increase while CD8^+^ T_EM_ cells decreased. Specifically, at day 188 post-primary vaccination (8 days post-boost), the frequency of IFN-γ^+^CD8^+^ T cells that were T_EMRA_ was 57 vs. 40% that were T_EM_. Together, these data indicate that DENV-specific CD8^+^ T cell response to TV003 is characterized by an early T_EM_ response that gradually gives rise to a long-lasting T_EMRA_ response.

### Natural History of DENV-Specific CD8^+^ and CD4^+^ T Cell and Neutralizing Antibody Responses in the Setting of Protective Vaccination in Humans

Previous studies from our team have demonstrated that DLAV vaccination provides protection against a subsequent DENV challenge in humans. In particular, the CIR287 study summarized in Figure 1B demonstrated that DLAV-vaccinated individuals were fully protected against the development of DENV viremia and rash when challenged 180 days after vaccination with the under-attenuated DENV2Δ30 (Tonga/74) (32). Thus, the CIR287 study provided us with a unique opportunity to evaluate both DENV-specific CD8^+^ and CD4^+^ T cells, as well as neutralizing antibodies, in the context of protective vaccination.

**Figure 5:**
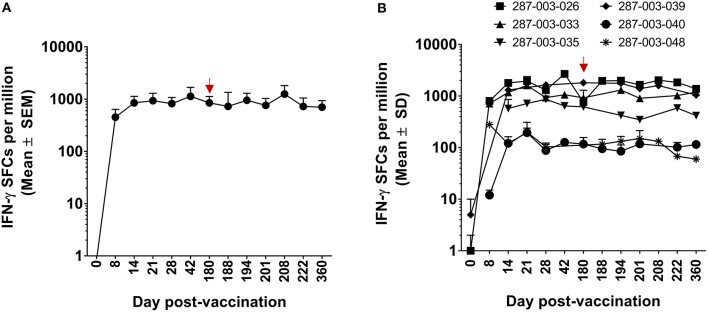
ELISPOT measurement of DENV-specific IFN-γ^+^CD8^+^ T cells in the setting of protective vaccination in CIR287 individuals. ELISPOT analysis was used to identify the number of IFN-γ producing CD8^+^ T cells that responded to the DENV CD8 MP following DLAV vaccination on day 0 and DENV2Δ30 (Tonga/74) challenge on day 180 post-vaccination. Note that the time of DENV2Δ30 (Tonga/74) challenge (day 180 post-vaccination) is shown in each graph with a red arrow. **(A)** Depicts the mean responses for selected individuals that were examined (*n* = 6) while **(B)** shows the individual response of these six donors.

**Figure 6:**
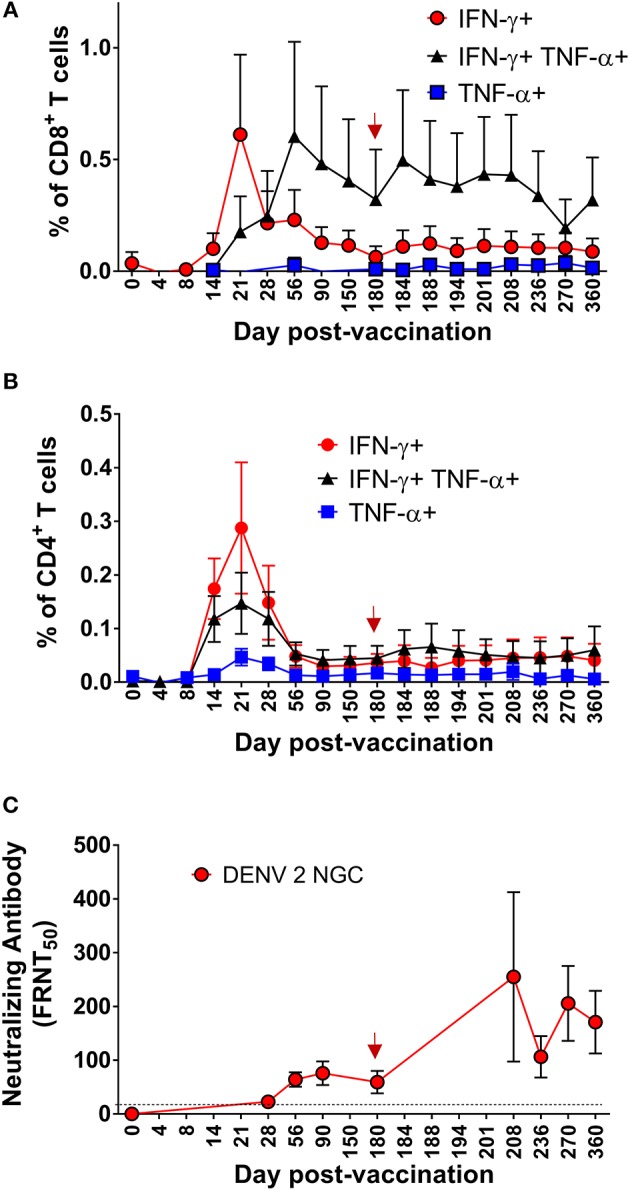
Kinetics of multifunctional CD8^+^ and CD4^+^ T cell or neutralizing antibody formation and maintenance in the setting of protective DENV vaccination in CIR287 individuals. Following DLAV vaccination on day 0 and DENV2Δ30 (Tonga/74) challenge on day 180 post-vaccination, ICS was used to measure the frequency of CD8^+^ T cells **(A)** or CD4^+^ T cells **(B)** that made IFN-γ, TNF-α, or both in response to the DENV CD8 MP **(A)** or DENV CD4 MP **(B)** while FRNT was used to measure the titer of anti-DENV2 NGC neutralizing antibodies **(C)**. Note that the time of DENV2Δ30 (Tonga/74) challenge (day 180 post-vaccination) is shown in each graph with a red arrow and that the limit of detection in **(C)** is indicated with a dashed line. For each patient sample, the background signal to DMSO was subtracted from the signal to the DENV CD8 MP **(A)** or DENV CD4 MP **(B)**. Mean values ± SEM are shown for the eight CIR287 individuals examined **(A–C)**.

We initially evaluated CD8^+^ T cell responses to the DENV CD8 MP via ELISPOT. As shown in Figures 5A,B, in six CIR287 donors, the kinetics and magnitude of the DENV-specific CD8^+^ T cells behaved similarly to that observed in the CIR268 cohort (Figure 2). Responses were detectable as early as day 8 post-vaccination, reached peak titers between 21 and 42 days after vaccination, and, for most donors, began to wane by day 180 post-vaccination. The magnitude of response varied between donors, but in all cases, responses were detectable throughout the first 180 days following vaccination. Similar to the CIR268 cohort, we observed accelerated kinetics of DENV-specific CD8^+^ T cells making IFN-γ or TNF-α, followed by the appearance of multifunctional CD8^+^ T cells making both IFN-γ and TNF-α (Figure 6A).

We next used ICS to measure the frequency and kinetics of DENV-specific CD4^+^ T cells from eight CIR287 vaccinees that responded to the DENV CD4 MP. Following vaccination, DENV-specific CD4^+^ T cells expressing IFN-γ, TNF-α, or both were detectable by day 14, peaked on day 21, decreased in frequency through day 56, and then remained relatively unchanged until day 180 (Figure 6B). Unlike the antiviral CD8^+^ T cells, multifunctional IFN-γ^+^TNF-α^+^ CD4^+^ T cells formed with the same kinetics as CD4^+^ T cells expressing only IFN-γ^+^ or TNF-α^+^.

While we did not screen for anti-DENV2 NGC neutralizing antibodies until day 28 post-vaccination, the eight CIR287 vaccinees examined in Figures 6A,B began to exhibit low levels of neutralizing antibodies on day 28 post-vaccination (Figure 6C). Antibody titers then increased and reached peak levels on day 90 post-vaccination and remained stable until day 180 post-vaccination. We observed no correlation between the magnitude of multi-functional CD4 and CD8 T cell responses on day 180 and antibody neutralization titers (data not shown). Thus, multifunctional CD4^+^ T cell formation and peak expansion (Figure 6B) occurs prior to the generation of antiviral neutralizing antibodies (Figure 6C) as well as multifunctional CD8^+^ T cells (Figure 6A).

**Figure 7:**
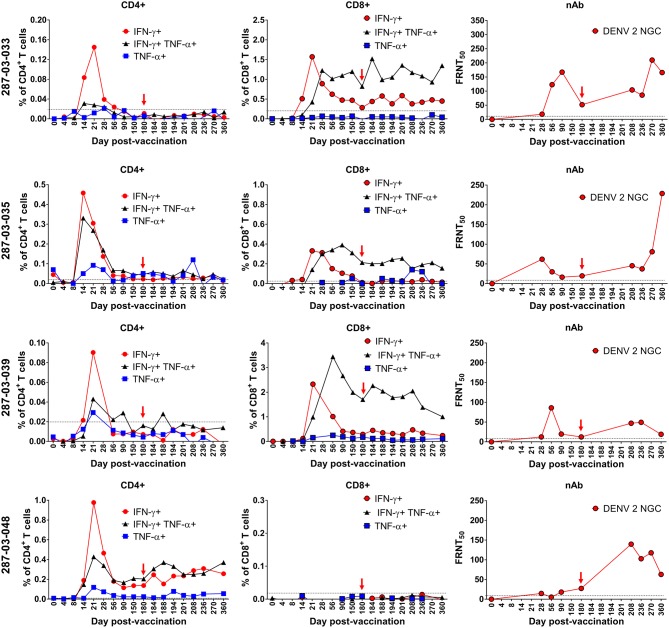
Kinetics of neutralizing antibody and multifunctional CD8^+^ and CD4^+^ T cell formation and maintenance in the setting of protective DENV vaccination in CIR287 individuals. ICS was used to measure the frequency of CD8^+^ T cells or CD4^+^ T cells that made IFN-γ, TNF-α, or both in response to the DENV CD8 MP or DENV CD4 MP, respectively, following DLAV vaccination on day 0 and DENV2Δ30 (Tonga/74) challenge on day 180 post-vaccination. Neutralizing antibody titer against DENV2 strain New Guinea C (NGC) was measured in the same individuals via the focus reduction neutralization (FRNT_50_) test. Note that in each graph the time of DENV2Δ30 (Tonga/74) challenge (day 180 post-vaccination) is shown with a red arrow and the limit of detection is indicated with a dashed line.

Similar to the ELISPOT analysis of CD8^+^ T cells from individual donors in Figures 2C, 5B, there was heterogeneity in the frequency and kinetics of individual CD8^+^ T cell, CD4^+^ T cell, and DENV2-specific neutralizing antibody responses. To illustrate this, Figure 7 shows the antiviral CD8^+^ T cell, CD4^+^ T cell, and DENV2 neutralizing antibody titers observed in representative donors (287-03-033, 287-03-035, 287-03-039, and 287-03-048). The findings from these individual donors are mostly consistent with the average trends seen when examining the mean values of the entire CIR287 cohort shown in Figure 6. There are several key observations from this analysis. First, there appears to be a relatively equal rate of IFN-γ^+^, TNF-α^+^, or IFN-γ^+^TNF-α^+^ CD4^+^ T cell formation. Further, at the peak of expansion on day 21 post-vaccination, IFN-γ^+^ CD4^+^ T cells reach higher frequencies when compared to TNF-α^+^ or IFN-γ^+^TNF-α^+^ CD4^+^ T cells. Second, there is a staggered appearance of IFN-γ^+^ and IFN-γ^+^TNF-α^+^ CD8^+^ T cells, with the IFN-γ^+^-only population forming earlier and the IFN-γ^+^TNF-α^+^ cells reaching peak levels later and remaining the highest frequency CD8^+^ T cell subset through 180 days post-vaccination. Last, neutralizing antibodies to DENV2 appear (and peak) after the establishment of DENV-specific CD8^+^ and CD4^+^ T cells.

### Impact of Virus Challenge on DENV-Specific CD8^+^ and CD4^+^ T Cells

Following challenge of CIR287 vaccinees with DENV2Δ30 (Tonga/74) at day 180 post-vaccination, virus-specific CD8^+^ and CD4^+^ T cell responses varied by donor. By CD8^+^ T cell ELISPOT, two donors showed increased DENV CD8^+^ T cells following challenge, while one showed a decline, and three remained unchanged (Figure 5B). By ICS, when mean values for the entire CIR287 cohort were examined, there appeared to be a trend of multifunctional IFN-γ^+^TNF-α^+^ CD4^+^ and CD8^+^ T cells increasing slightly on day 184 following vaccination (day 4 post-challenge) and then either maintaining at this frequency or decreasing through day 360 post-vaccination (d180 post-challenge) (Figures 6A,B). When examined at the individual level, the vaccinees shown in Figure 7 did not show appreciable boosting of DENV-specific CD4^+^ T cells by DENV2Δ30 (Tonga/74) challenge, with the possible exception of donor 287-03-035. However, this analysis is complicated by the fact that CD4^+^ T cell reactivity was below the limit of detection for all three donors at most time points following challenge. With regard to DENV-specific CD8^+^ T cells, two of the three donors (287-03-033 and 287-03-039) shown in Figure 7 had increases in IFN-γ^+^TNF-α^+^ cells following challenge with DENV2Δ30 (Tonga/74) while donor 287-03-035 maintained even cell frequencies directly after challenge.

### Memory CD8^+^ and CD4^+^ T Cell Populations in the Setting of Protective DENV Vaccination

**Figure 8:**
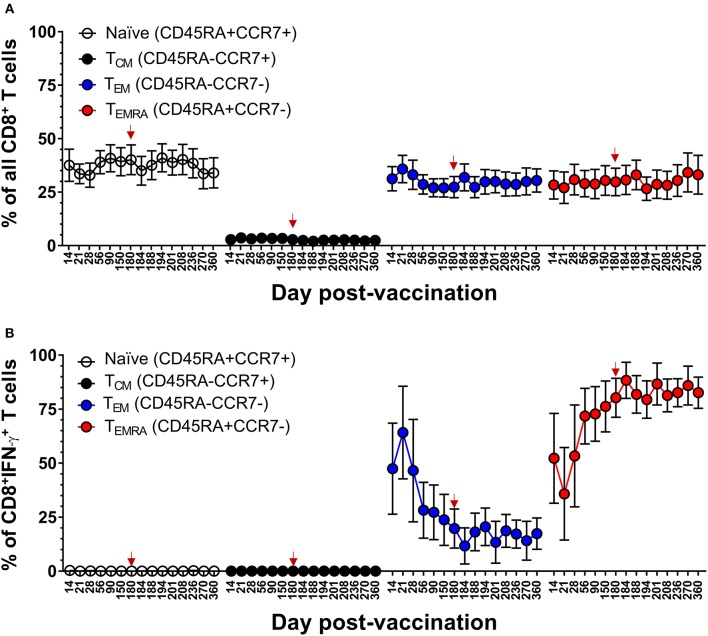
Memory CD8^+^ T cell populations in the setting of protective DENV vaccination in CIR287 individuals. **(A)** ICS was used to measure the frequency of all CD8^+^ T cells exhibiting different memory T cell phenotypes (naïve, CD45RA^+^CCR7^+^; T_CM_, CD45RA^−^CCR7^+^; T_EM_, CD45RA^−^CCR7^−^; or T_EMRA_, CD45RA^+^CCR7^−^) in CIR287 vaccinees following DLAV vaccination on day 0 and DENV2Δ30 (Tonga/74) challenge on day 180 post-vaccination. **(B)** Shows the frequency of IFN-γ^+^CD8^+^ T cells that exhibited different memory phenotypes following stimulation with the DENV CD8 MP. Mean values ± SEM are shown for the eight CIR287 individuals examined. Note that the time of DENV2Δ30 (Tonga/74) challenge (day 180 post-vaccination) is shown in each graph with a red arrow.

We next examined the kinetics and phenotypic profile of DENV-specific memory CD8^+^ and CD4^+^ T cells that were elicited by DLAV vaccination in the CIR287 cohort and associated with complete protection against DENV2Δ30 (Tonga/74) challenge. CIR287 individuals exhibited a similar pattern of memory CD8^+^ T cell formation to that seen for the CIR268 vaccinees in Figure 4. Specifically, the initial virus-specific CD8^+^ T cell response on days 14–21 following vaccination was dominated by T_EM_ cells in CIR287 donors (Figure 8B) (Note that Figure 8A shows memory subset frequencies for CD8^+^ T cells unable to elicit IFN-γ in response to CD8 megapool stimulation). Thereafter, the frequency of CD8^+^ T_EM_ cells steadily declined and returned to baseline levels. Conversely, the frequency of CD8^+^ T_EMRA_ cells steadily increased from day 21 after vaccination to day 180 where it represented 68% of virus-specific IFN-γ^+^CD8^+^ T cells. Following DENV2Δ30 (Tonga/74) challenge, CD8^+^ T_EMRA_ cells continued to increase for 8 days, then declined slightly and became stable whereas CD8^+^ T_EM_ cells showed a slight increase on day 4 post-challenge (day 184 post-vaccination), and then remained fairly stable through day 180 post-challenge (day 360 post-vaccination).

**Figure 9:**
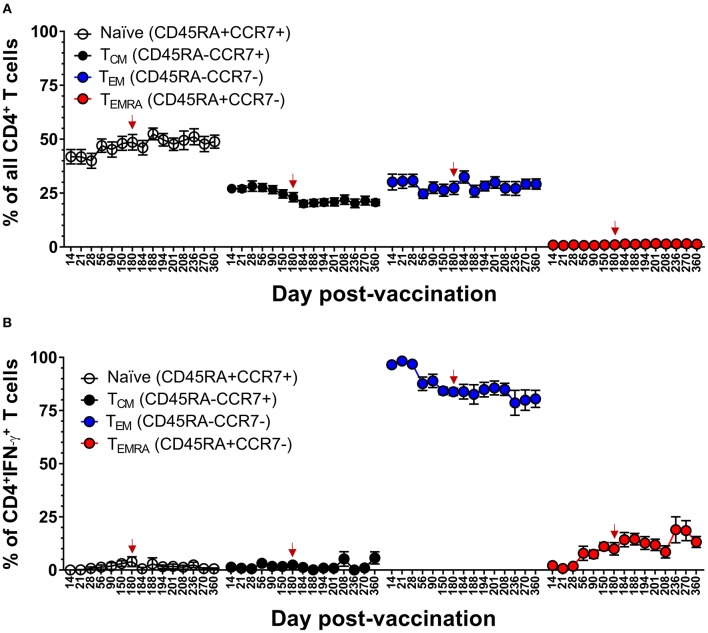
Memory CD4^+^ T cell populations in the setting of protective DENV vaccination in CIR287 individuals. **(A)** ICS was used to measure the frequency of all CD4^+^ T cells exhibiting different memory T cell phenotypes (naïve, CD45RA^+^CCR7^+^; T_CM_, CD45RA^−^CCR7^+^; T_EM_, CD45RA^−^CCR7^−^; or T_EMRA_, CD45RA^+^CCR7^−^) in CIR287 vaccinees following DLAV vaccination on day 0 and DENV2Δ30 (Tonga/74) challenge on day 180 post-vaccination. **(B)** Shows the frequency of IFN-γ^+^CD4^+^ T cells that exhibited different memory phenotypes following stimulation with the DENV CD4 MP. Mean values ± SEM are shown for the eight CIR287 individuals examined. Note that the time of DENV2Δ30 (Tonga/74) challenge (day 180 post-vaccination) is shown in each graph with a red arrow.

We had previously reported that DLAV vaccination induces virus-specific CD4^+^ T cells that are predominantly T_EM_ at 10–26 months after vaccination (20). Here, we confirm and extend this observation by demonstrating that while the majority of DENV-specific memory CD4^+^ T cells were T_EM_ (range 78–98% of IFN-γ^+^CD4^+^ T cells), vaccination induces a progressive increase in the DENV-specific CD4^+^ T_EMRA_ cells over 180 days, and that this frequency is maintained for at least 180 days after challenge with DENV (Tonga/74) (Figure 9). DENV-specific CD4^+^ T_EMRA_ cells were initially very low between days 14 and 28 post-vaccination (range 1 to 2% of IFN-γ^+^CD4^+^ T cells) and then steadily increased to ~7–19% of IFN-γ^+^CD4^+^ T cells. Following challenge, there appeared to be a slight trend of gradually increasing antiviral CD4^+^ T_EMRA_. Taken together, these data suggested that protective immunity against DENV in a human challenge model was associated with an early effector phase marked by the generation of multi-functional CD4^+^ and CD8^+^ T_EM_ cells, followed by late phase marked by a progressive increase in the frequency of CD4^+^ and CD8^+^ T_EMRA_ cells.

## Discussion

DENV is a serious threat to human health and the current lack of an FDA-approved vaccine for dengue-naïve individuals to safely prevent disease from all four DENV serotypes is a major unmet medical need. Further, the immunological basis for protective immunity to DENV infection is not fully understood. Certainly there is support that both arms of the adaptive immune response, T and B cells, play an important role (45, 46). In the current study, we had the opportunity to detail the natural history and functional attributes of DENV-specific CD8^+^ and CD4^+^ T cells in the setting of protective DENV vaccination and to view these T cell responses concurrently with antiviral neutralizing antibodies. There were several key findings. First, multifunctional (e.g., IFN-γ^+^TNF-α^+^-producing) CD8^+^ and CD4^+^ T cells specific for DENV form rapidly, typically within the first 8–14 days after vaccination and remain detectable for at least 6 months. Second, multifunctional CD4^+^ T cells form prior to both multifunctional CD8^+^ T cells and antiviral neutralizing antibodies and thus may contribute the establishment and quality of these CD8^+^ T cell and antibody responses. Third, vaccine-induced CD8^+^ T cells that are dominated by T_EM_ early after vaccination eventually give way to increased frequencies of T_EMRA_ cells that remain elevated 1 year after vaccination. Last, although the majority of DENV-specific CD4^+^ T cells induced by DLAV vaccination are T_EM_, and only a small proportion are T_EMRA_, the frequency of virus-specific CD4^+^ T_EMRA_, is significantly increased after vaccination and challenge. Thus, our study details for the first time the formation, maintenance, and phenotypic profile of antiviral CD8^+^ and CD4^+^ T cells associated with protection against DENV infection.

There are several lines of evidence to suggest that DENV-specific CD8^+^ and CD4^+^ T cell responses play a protective role against DENV infection and/or disease severity (18, 21, 33–42). Indeed, data from the CIR287 study (DLAV vaccination followed by DENV2Δ30 (Tonga/74) challenge) adds support to this hypothesis. Specifically, of the 21 DLAV vaccinees who were protected from DENV2Δ30 (Tonga/74) challenge, nine exhibited a 4-fold or greater boost in their antiviral neutralizing antibodies following challenge (32). This result indicates that sterilizing immunity from neutralizing antibodies was not the sole mechanism of protection at work in these individuals. Rather, it is possible that the multifunctional CD8^+^ and/or CD4^+^ T cells detected in the CIR287 vaccinees contributed to the observed protection. However, formally demonstrating that DLAV-induced CD8^+^ and/or CD4^+^ T cells are sufficient to protect humans against DENV infection remains a challenge considering the high rate of neutralizing antibody induction typically observed in vaccinees (31, 32).

Our team previously demonstrated that DLAV vaccination elicits virus-specific CD8^+^ and CD4^+^ T cells recognizing all four DENV serotypes with the same antigen specificity and phenotypic attributes as those formed during natural DENV infection (19, 20). Further, these DLAV-induced CD8^+^ and CD4^+^ T cell responses remain detectable for at least 12 or 26 months, respectively (19, 20). In the current study, we were able to fine map the appearance of DENV-specific CD8^+^ and CD4^+^ T cells following vaccination. Thus, assuming that these T cells are protective, our studies collectively suggest that DLAV vaccination may provide protection within 8–14 days and that this protection could last for at least a year. The kinetics and phenotype of CD8^+^ T cell induction and maintenance in humans has also been examined following vaccination with Takeda's live-attenuated tetravalent dengue vaccine (TDV) consisting of an attenuated DENV2 strain (TDV-2), and three chimeric viruses encoding the pre-membrane (prM) and E proteins of DENV1, 2, or 4 on the TDV-2 backbone. Chu et al. examined DENV-specific CD8^+^ T cells on days 14 and 90 after primary vaccination and, by ICS, could detect multifunctional (IFN-γ^+^TNF-α^+^) CD8^+^ T cells at the day 90 time point (47). Subjects received a boost at this same time point (day 90 post-primary vaccination) and retained DENV-specific CD8^+^ T cells for another 90 days. More recently, Waickman et al. detected DENV-specific CD8^+^ T cells by IFN-γ ELISPOT as early as 28 days following administration of this same live-attenuated tetravalent dengue virus vaccine candidate (48). Thus, there are similarities in CD8^+^ T cell responses elicited by the DLAV and TDV platforms and our studies help to more precisely fill in the timing of anti-DENV T cell formation and maintenance following vaccination. Future human challenge studies will be required to define how quickly protective immunity is established following vaccination and the durability of this protective response.

A considerable challenge to the development of a safe DENV vaccine has been the requirement to simultaneously induce protective immunity to all four DENV serotypes. Failure to do so theoretically puts vaccinees at risk of developing severe DENV disease due to antibody-dependent enhancement, a phenomenon whereby antiviral antibodies raised against one serotype (e.g., DENV1) can bind a second serotype (e.g., DENV3) and lead to enhanced entry of this virus into target cells (7). Indeed, the underperformance of Dengvaxia (9–14), a tetravalent DENV vaccine with the prM and E proteins of DENV and the backbone of yellow fever virus, illustrates the possible danger of a vaccine that primarily targets the generation of neutralizing antibodies, but not antiviral T cells. Not only has Dengvaxia failed to fully protect against DENV infection, it increases the risk of hospitalization in DENV-naïve individuals when compared to unvaccinated individuals (9, 15, 16). One possible advantage of a live-attenuated vaccine like DLAV is that it induces balanced CD8^+^ and CD4^+^ T cells responses to all four DENV serotypes after a single dose, with a particular focus on several of the DENV non-structural proteins that are missing from Dengvaxia (17–21). It is a possibility that the multifunctional CD8^+^ and CD4^+^ T cells induced by DLAV may not only provide protection against primary DENV infection, but could also counteract the more severe DENV disease caused by antibody-dependent enhancement.

The generation of CD8^+^ T cell memory after vaccination is associated with progressive changes in the frequencies of virus-specific T_EM_ and T_EMRA_ cells (49, 50). Previous studies have demonstrated that long-term CD8^+^ and CD4^+^ T cell memory following both natural DENV infection and DLAV vaccination is associated with multi-functional T_EM_ and/or T_EMRA_ cells (19, 20, 51). Our results here reveal the dynamics of the formation and maintenance of these memory T cell populations in the setting of a protective immune response to dengue virus (32). For both CD4^+^ and CD8^+^ T cells, the generation of multifunctional T cells in the first 2–4 weeks after vaccination is associated primarily with a T_EM_ phenotype, after which there is a steady increase in the frequency of virus-specific T_EMRA_ cells until 180 days after vaccination. These kinetics are similar to those previously observed after vaccination with both yellow fever and smallpox (52), indicating that these phenotypic changes are not restricted to specific pathogens. Rather, they are phenotypic features associated with the generation of virus-specific T cell memory. Indeed, previous reports that a high frequency of CD8^+^ T_EMRA_ is associated with protection against symptomatic H1N1 influenza (53) and HSV-1 reactivation (52) underscore the relevance of using T_EMRA_ generation as a primary goal in the design of effective vaccines.

The mechanisms underlying the efficacy of T_EMRA_ in the memory response remain unclear. We found that although the initial response to DLAV is dominated by T_EM_ cells, the virus-specific response upon dengue challenge or DLAV boost is dominated by T_EMRA_ cells, indicating that it may be the T_EMRA_ subset that drives the memory T cell immune response. This finding is consistent with a recent report indicating that CD8^+^ T_EMRA_ cells retain epigenetic marks that foster rapid effector function (50). Although comparatively less is known of CD4^+^ T_EMRA_, it was recently shown that DENV-specific CD4^+^ T_EMRA_ cells are cytolytic and are associated with protective immunity (41, 54, 55).

In conclusion, these data provide a detailed map of the natural history of DENV-specific CD4^+^ and CD8^+^ T cell phenotype and function in a human challenge model of protective DLAV vaccination. Our data demonstrate that the protective DLAV vaccine elicits multi-functional CD4^+^ and CD8^+^ T_EMRA_ cells and suggest that these virus-specific T cells may play a role in protective immunity. Future studies will be needed to determine whether these DENV-specific T cell populations are a bona fide correlate of protection against DENV infection.

## Data Availability Statement

All datasets generated for this study are included in the article/Supplementary Material.

## Ethics Statement

The studies involving human participants were reviewed and approved by Institutional Review Boards at the University of Vermont and Johns Hopkins University. The patients/participants provided their written informed consent to participate in this study.

## Author Contributions

AS, DW, JEB, and JWB conceived and designed the experiments. NG, PE, and DW performed the experiments. KP, BK, AD, SD, and SW conducted the clinical trials at UVM and JHU, and provided the specimens. NG, PE, JEB, and JWB analyzed the data. BK, JEB, and JWB wrote the manuscript and all co-authors participated in the editorial process and approved the manuscript.

### Conflict of Interest

The authors declare that the research was conducted in the absence of any commercial or financial relationships that could be construed as a potential conflict of interest.
